# Supplementary material for: Content-rich biological network constructed by mining PubMed abstracts
Source: BMC Bioinformatics. 2004 Oct 8;5:147. doi: 10.1186/1471-2105-5-147 (PMC528731; doi:10.1186/1471-2105-5-147)
Supplement: Additional File 2 — The original results of the above study (non-essential files are deleted to keep the file size under the limit set by BMC bioinformatics). [file 1471-2105-5-147-S2.bz2 › chilibotAdditionalFile2/dip30/549ID7910967E10902/html/CD4_IL16.html]

 


 **CD4** and **IL16** 
  
Found 142 abstracts in PubMed, retrieved 30.  
 

 What does Google say? 
 PDF only 
| .edu only 

---

**Interactive relationship** (e.g. stimulation, inhibition, etc)

**Inhibitory relationship**- interleukin IL 16  [ **IL16** ]  can inhibit the activity of  **CD4**  T lymphocytes.  Ref: 12869037 Clin Exp Immunol, 2003
- BACKGROUND IL 16  [ **IL16** ]  has been described as a natural soluble  **CD4**  ligand with immunosuppressive effects in vitro.  Ref: 12569988 Clin Exp Allergy, 2002
- Agonists selective for H 2 dimaprit, H 3 4 R alpha methylhistamine, and H 4 clobenpropit were capable of inducing the release of bioactive IL 16  [ **IL16** ]  because CD8 cell supernatants induced  **CD4**  cell migration, which was abrogated by an anti IL 16  [ **IL16** ]  antibody.  Ref: 12235264 J Pharmacol Exp Ther, 2002
- The decrease of  **CD4**  T cells in MM may be mediated by activation of CD8 T cells derived cytokine IL 16  [ **IL16** ] .  Ref: 12191564 Leuk Res, 2002
- The resulting cells responded to IL 16  [ **IL16** ]  in an in vitro chemotaxis assay, and this biologic response could be blocked by anti IL 16  [ **IL16** ]  and anti  **CD4**  Abs as well as by a competitive peptide inhibitor corresponding to a sequence in the C terminal domain of IL 16  [ **IL16** ] .  Ref: 11937573 J Immunol, 2002
**Neutral relationship**- IL 16  [ **IL16** ]  is a  **CD4**  specific ligand, and RANTES is a C C type chemokine that targets monocytes and lymphocytes.  Ref: 12810540 Endocrinology, 2003
- IL 16  [ **IL16** ]  is a ligand and chemotactic factor for  **CD4**  T cells.  Ref: 12528894 Cell Res, 2002
- In accordance with other data in the medical literature, we conclude that the most important function of IL 16  [ **IL16** ]  is the chemoattraction of  **CD4**  cells.  Ref: 12001788 Allergy Asthma Proc< MedlineTA>Allergy Asthma Proc, 2002
- **CD4**  serves as a coreceptor for HIV and IL 16  [ **IL16** ] .  Ref: 12213222 Exp Cell Res, 2002
- CONCLUSIONS IL 16  [ **IL16** ]  is involved in the asthmatic process by recruiting  **CD4**  T cells and thus plays an important role in  **CD4**  cell dependent eosinophilic inflammation in asthmatic airways.  Ref: 11775918 Zhonghua Jie He He Hu Xi Za Zhi, 1999
- Here we show that these effects of  **CD4**  ligation were not observed when anti  **CD4**  antibody was replaced with another  **CD4**  ligand, interleukin 16  [ **IL16** ] , or when the anti CD3 and anti  **CD4**  antibodies were spatially separated by immobilization on different beads.  Ref: 12848849 Immunol Cell Biol< MedlineTA>Immunol Cell Biol, 1999
- Interleukin 16  [ **IL16** ]  IL 16  [ **IL16** ]  has been described as a main cytokine involved in  **CD4**  cell recruitment during inflammation.  Ref: 12169178 Allergy, 2002
- It was also found that the percentages of  **CD4**  and EG2 cells were all positively correlated to IL 16  [ **IL16** ]  levels.  Ref: 11775918 Zhonghua Jie He He Hu Xi Za Zhi, 1999
- Recently the influx of  **CD4**  lymphocytes has been related to the up regulation of IL 16  [ **IL16** ]  in AEDS skin lesions.  Ref: 12169178 Allergy, 2002
- The first of these, interleukin 16  [ **IL16** ] , is an immunomodulatory cytokine involved in the regulation of  **CD4**  T cells.  Ref: 11967309 J Virol, 2002
- Interleukin 16  [ **IL16** ]  IL 16  [ **IL16** ]  is a cytokine which is mainly produced by CD8 T cells and induces chemotaxis of  **CD4**  T cells and monocytes.  Ref: 11999883 Lupus, 2002
- IL 16  [ **IL16** ] , IL 8, IP 10 and RANTES are participated in the accumulation of  **CD4**  T cell.  Ref: 12233067 Nippon Rinsho, 2002
- IL 16  [ **IL16** ]  plays a role in trafficking of several immune cells and may be a major chemotactic signal for  **CD4**  cells.  Ref: 12001788 Allergy Asthma Proc< MedlineTA>Allergy Asthma Proc, 2002
- IL 16  [ **IL16** ]  expression in LCs in patients with AD correlated with the number of infiltrating  **CD4**  cells r =.72, P =.0017 and was completely downregulated parallel to the clinical response of AD lesions to topical treatment with FK506.  Ref: 11941319 J Allergy Clin Immunol, 2002
- Therefore, IL 16  [ **IL16** ]  activation of eosinophils is  **CD4**  mediated to elicit the extracellular release of preformed RANTES and eotaxin, which then in an autocrine fashion act on plasma membrane CCR3 receptors to stimulate both enhanced LTC 4 production and the preferential release of IL 4, but not IL 12, from within eosinophils.  Ref: 11971026 J Immunol, 2002
- Recently, epidermal cells in lesional AD were reported to contain increased messenger m RNA levels of IL 16  [ **IL16** ] , a cytokine that induces chemotactic responses in  **CD4**  T cells, monocytes, and eosinophils.  Ref: 11941319 J Allergy Clin Immunol, 2002
- **CD4**  is a recognized IL 16R, and accordingly anti  **CD4**  Fab, soluble  **CD4** , and a  **CD4**  domain 4 based IL 16  [ **IL16** ]  blocking peptide inhibited the actions of IL 16  [ **IL16** ]  on eosinophils.  Ref: 11971026 J Immunol, 2002

**Non-interactive relationship** (e.g. studied together, co-existance, homology, etc.)

- MIP 1beta induced G protein activation was further increased by simultaneous stimulation of  **CD4**  with its natural agonist, interleukin 16  [ **IL16** ] .  Ref: 12531905 J Biol Chem, 2003
- The modulation of immune cell costimulatory molecules, CD80, CD86, CD40, and HLA DR, and the release of the  **CD4**  T cell chemoattractant IL 16  [ **IL16** ]  were also investigated.  Ref: 12804531 Clin Immunol, 2003
- R5 tropic HIV 1 glycoprotein 120, but not interleukin 16  [ **IL16** ] , the natural agonist, or X4 tropic glycoprotein 120, inhibited MIP 1beta binding to CCR5 in the presence of monomeric and dimeric soluble  **CD4** .  Ref: 12878220 Biochem Biophys Res Commun, 2003
- As IL 16  [ **IL16** ]  has been shown to regulate  **CD4**  cell recruitment and activation, and is detected early following airway challenge of individuals with asthma, this two step process for IL 16  [ **IL16** ]  production by epithelial cells may represent a rapid response mechanism in the orchestration of allergic airway inflammation.  Ref: 12594062 Am J Respir Cell Mol Biol, 2003
- Furthermore, Mo and AM were separately exposed to the three PM size fractions and supernatants assessed for the T helper  **CD4**  lymphocyte chemoattractant interleukin 16  [ **IL16** ]  IL 16  [ **IL16** ] .  Ref: 12746131 J Toxicol Environ Health A, 2003
- Although  **CD4**  is not G protein coupled, pertussis toxin inhibited IL 16  [ **IL16** ]  induced eosinophil activation.  Ref: 11971026 J Immunol, 2002
- We reported previously that fibroblasts from patients with GD express IL 16  [ **IL16** ] , a  **CD4**  specific chemoattractant, and RANTES, a C C chemokine, in response to GD specific IgG GD IgG.  Ref: 12794168 J Immunol, 2003
- IL 16  [ **IL16** ]  is a  **CD4**  specific chemoattractant and RANTES is a C C type chemokine.  Ref: 11777993 J Immunol, 2002
- AIMS To examine if IL 16  [ **IL16** ] , a chemoattractant for  **CD4**  cells, is detectable in airway secretions of ventilated newborns.  Ref: 11893439 Early Hum Dev, 2002
- Relationship between  **CD4**  CD8 T cell ratio and T cell activation in multiple myeloma reference to IL 16  [ **IL16** ] .  Ref: 12191564 Leuk Res, 2002
- Because IL 16  [ **IL16** ]  is a ligand for  **CD4**  and or an undefined  **CD4**  associated protein, the ability of this multifunctional cytokine to regulate the development of human MCs basophils from nongranulated progenitors residing in cord or peripheral blood was evaluated.  Ref: 11937573 J Immunol, 2002
- BACKGROUND It is currently unknown if interleukin IL 16  [ **IL16** ]  exists in the lungs of ventilated infants, and because the predominant cells in the airways of infants with CLD are  **CD4**  macrophages, we hypothesized that IL 16  [ **IL16** ]  plays a role as a pro inflammatory mediator in lung inflammation.  Ref: 11893439 Early Hum Dev, 2002
- IL 16  [ **IL16** ]  promotes leukotriene C 4 and IL 4 release from human eosinophils via  **CD4**  and autocrine CCR3 chemokine mediated signaling.  Ref: 11971026 J Immunol, 2002
- ELISA was used to determine the concentrations of IL 16  [ **IL16** ]  in sputum, and  **CD4**  T cells and activated eosinophils EG2 cells in sputum were detected by immunostaining.  Ref: 11775918 Zhonghua Jie He He Hu Xi Za Zhi, 1999
